# HOME AND COMMUNITY-BASED CARE FOR A DIVERSE AND GROWING POPULATION: WORKFORCE POLICY AND PRACTICE IMPLICATIONS

**DOI:** 10.1093/geroni/igz038.1656

**Published:** 2019-11-08

**Authors:** Kezia Scales, Jodi M Sturgeon, Lisa I Iezzoni, Robert Espinoza, Stephen Campbell, Allison Cook, Naomi I Gallopyn

**Affiliations:** 1 PHI, Bronx, New York, United States; 2 Health Policy Research Center, Mongan Institute, Massachusetts General Hospital, Boston, Massachusetts, United States; 3 PHI (Paraprofessional Healthcare Institute), Bronx, New York, United States

## Abstract

Most Americans would prefer to continue living in their homes and communities as they age, even when they require support with daily activities due to illness or disability. Much of this support is provided by unpaid caregivers, but the paid home care workforce also plays an essential role. Due to demographic changes and poor job quality, however, the home and community-based services (HCBS) sector is struggling to attract and retain enough workers to meet demand. Drawing from an extensive analysis of HCBS in the United States, this paper examines key factors impacting the home care workforce, including: supply and demand trends; financing policies; service-delivery models; and policies and practices defining workers’ compensation, training, and career development. From these findings, we provide recommendations for addressing the home care workforce crisis and maximizing home care workers’ contribution to the delivery of high-quality supports for a growing and evolving population of HCBS consumers.

